# Both cardiomyocyte and endothelial cell Nox4 mediate protection against hemodynamic overload-induced remodelling

**DOI:** 10.1093/cvr/cvx204

**Published:** 2017-10-13

**Authors:** Min Zhang, Heloise Mongue-Din, Daniel Martin, Norman Catibog, Ioannis Smyrnias, Xiaohong Zhang, Bin Yu, Minshu Wang, Ralf P Brandes, Katrin Schröder, Ajay M Shah

**Affiliations:** 1 Cardiovascular Division, James Black Centre, King’s College London British Heart Foundation Centre of Excellence, 125 Coldharbour Lane, London SE5 9NU, UK;; 2 Institut für Kardiovaskuläre Physiologie, Goethe-Universität, 60590 Frankfurt am Main, Germany

**Keywords:** Cardiac remodelling, NADPH oxidase, Nox4, Reactive oxygen species

## Abstract

**Aims:**

NADPH oxidase-4 (Nox4) is an important reactive oxygen species (ROS) source that is upregulated in the haemodynamically overloaded heart. Our previous studies using global Nox4 knockout (Nox4KO) mice demonstrated a protective role of Nox4 during chronic abdominal aortic banding, involving a paracrine enhancement of myocardial capillary density. However, other authors who studied cardiac-specific Nox4KO mice reported detrimental effects of Nox4 in response to transverse aortic constriction (TAC). It has been speculated that these divergent results are due to cell-specific actions of Nox4 (i.e. cardiomyocyte Nox4 detrimental but endothelial Nox4 beneficial) and/or differences in the model of pressure overload (i.e. abdominal banding vs. TAC). This study aimed to (i) investigate whether the effects of Nox4 on pressure overload-induced cardiac remodelling vary according to the pressure overload model and (ii) compare the roles of cardiomyocyte vs. endothelial cell Nox4.

**Methods and results:**

Global Nox4KO mice subjected to TAC developed worse cardiac remodelling and contractile dysfunction than wild-type littermates, consistent with our previous results with abdominal aortic banding. Next, we generated inducible cardiomyocyte-specific Nox4 KO mice (Cardio-Nox4KO) and endothelial-specific Nox4 KO mice (Endo-Nox4KO) and studied their responses to pressure overload. Both Cardio-Nox4KO and Endo-Nox4KO developed worse pressure overload-induced cardiac remodelling and dysfunction than wild-type littermates, associated with significant decrease in protein levels of HIF1α and VEGF and impairment of myocardial capillarization.

**Conclusions:**

Cardiomyocyte as well as endothelial cell Nox4 contributes to protection against chronic hemodynamic overload-induced cardiac remodelling, at least in part through common effects on myocardial capillary density.

## 1. Introduction

Increased production of reactive oxygen species (ROS) is involved in cardiac responses to hemodynamic stress but different ROS sources have varying roles. Unlike sources such as mitochondria, uncoupled nitric oxide synthases, and xanthine oxidases, the NADPH oxidase (Nox) family of enzymes produce ROS as their primary function.^1^ Nox proteins are especially important in redox signalling as opposed to non-specific oxidative damage.^2^ Of the seven known mammalian Nox isoforms (Nox1-5 and Duox1-2), Nox2 (also known as gp91^phox^) and Nox4 are the major ones in the heart. 

Overwhelming evidence indicates that Nox2 is involved in the development of cardiac hypertrophy, remodelling, arrhythmia, myocyte death, and fibrosis induced by diverse disease stimuli.^3^ The role of Nox4 in the heart, however, is controversial. Using mice with a global knockout of Nox4 (Nox4KO) and cardiomyocyte-targeted *Nox4*-overexpressing mice, we previously reported that Nox4 is protective against pressure overload-induced cardiac remodelling and dysfunction induced by chronic abdominal aortic banding (AAB).^4^ Cardiomyocyte Nox4 promoted a paracrine preservation of myocardial capillary density through enhanced hypoxia-inducible factor 1 (HIF1) and vascular endothelial growth factor (VEGF) signalling,^4^ an effect known to ameliorate hemodynamic overload-induced remodeling.^5^ However, Sadoshima’s group reported that an independently generated line of cardiomyocyte-specific Nox4KO mice showed less remodelling and dysfunction than wild-type (WT) mice in response to transverse aortic constriction (TAC), and concluded that Nox4 was detrimental in this context.^6^ Since our initial paper, a Nox4-dependent beneficial enhancement of angiogenesis and HIF signalling (similar to our findings in heart) has been confirmed in other organs by several groups.^7–9^ Furthermore, the potential for Nox4 to mediate protective signalling in the heart and vasculature has also been demonstrated by several independent studies.^10–15^ However, whether Nox4 mediates protective effects during chronic cardiac overload has remained widely debated. It has been speculated that the divergent results above reflect differences in the model of pressure overload model (AAB vs. TAC) and/or that cardiomyocyte Nox4 may be detrimental but endothelial Nox4 is beneficial.

In this study, we specifically investigated whether the beneficial effect of Nox4 previously observed during chronic AAB is also found in response to chronic TAC. We then compared the cell-specific roles of cardiomyocyte and endothelial cell Nox4. Our results conclusively demonstrate that both cardiomyocyte and endothelial Nox4 are capable of mediating protection against chronic pressure overload-induced cardiac remodelling in the mouse heart, at least in part through common mechanisms. We also report that Nox4-dependent beneficial effects are manifest during TAC as well as AAB.

## 2. Methods

### 2.1 Animal studies

All procedures were approved under the ‘Guidance on the Operation of the Animals’ (Scientific Procedures) Act, 1986 (UK Home Office) and by the institutional ethics committee. To generate inducible cardiomyocyte-specific Nox4KO mice (Cardio-Nox4KO), Nox4^fl/fl^ female mice^4^ were crossed with male α-MHC-MerCreMer mice.^16^ Tamoxifen was administered by ip injection (20 mg/kg/day) for 5 days in 3-week-old mice to induce Cre expression. Cardiomyocyte-specific targeting of Nox4 was confirmed by PCR as well as by Western blot analysis in isolated cardiomyocytes.^17^ Endothelial-specific Nox4KO mice (Endo-Nox4KO) were generated by crossing Nox4^fl/fl^ female mice with Tie2-Cre males.^18^ Global Nox4KO mice were described previously.^4^ All lines were back-crossed  >10 generations onto a C57BL/6 background.

Minimally invasive TAC was performed in 10- to 12-week old male Nox4KO mice and WT littermate controls under 2% isoflurane anaesthesia, without sternotomy or ventilation as described previously.^19^ Aortic constriction was performed by ligation of the transverse thoracic aorta with a 27-gauge needle using a 6-0 braided polyester suture. Mice were studied 2 weeks after TAC. Supra-renal AAB was performed in male animals (body weight 16–18 g) for 6 weeks as previously described.^4^ Cardio-Nox4KO were compared with tamoxifen-treated α-MHC-MerCreMer mice and with Nox4^fl/fl^ controls. Endo-Nox4KO mice were compared with Nox4^fl/fl^ controls. Cardiac structure and function were assessed by echocardiography using a Vevo2100 system (Visualsonics, Toronto, Canada).^4^^,^^20^

### 2.2 Histology

Animals were euthanized by cervical dislocation prior to harvesting of tissues. Interstitial fibrosis and myocardial capillary density were assessed in left ventricular (LV) sections.^4^ Deletion of endothelial Nox4 was identified by immunofluorescence in cryosections of the aorta.

### 2.3 Western blotting

Snap-frozen heart tissue samples or pelleted cardiomyocytes were homogenized and lysed in RIPA lysis buffer. To examine HIF1α protein, heart samples were rapidly homogenized in a buffer containing 4 M urea, 140 mM Tris (pH 6.8), 1% SDS, 2% NP-40, and protease inhibitors (Roche, Grenzach, Germany).^21^ For immunoblot analysis, protein samples were resolved by SDS-PAGE and transferred onto nitrocellulose membranes. The following antibodies were used: Nox2 and eNOS (BD Biosciences, Wokingham, UK); Nox4^4^; HIF1α (Novus , Abingdon, UK); VEGF and p-eNOS (p-S1177; Abcam, Cambridge, UK). Actin or α-actinin (Sigma, Gillingham, UK) were used as a loading control. Blots were quantified by densitometry.

### 2.4 Statistics

Data are expressed as mean ± SEM. Comparisons were undertaken on GraphPad Prism 5.0 by Student’s *t*-test or two-way ANOVA followed by Tukey’s *post hoc* analysis to compare groups as appropriate. *P *<* *0.05 was considered significant.

## 3. Results

### 3.1 Nox4KO mice develop exaggerated cardiac hypertrophy and dysfunction after TAC

**Figure 1:**
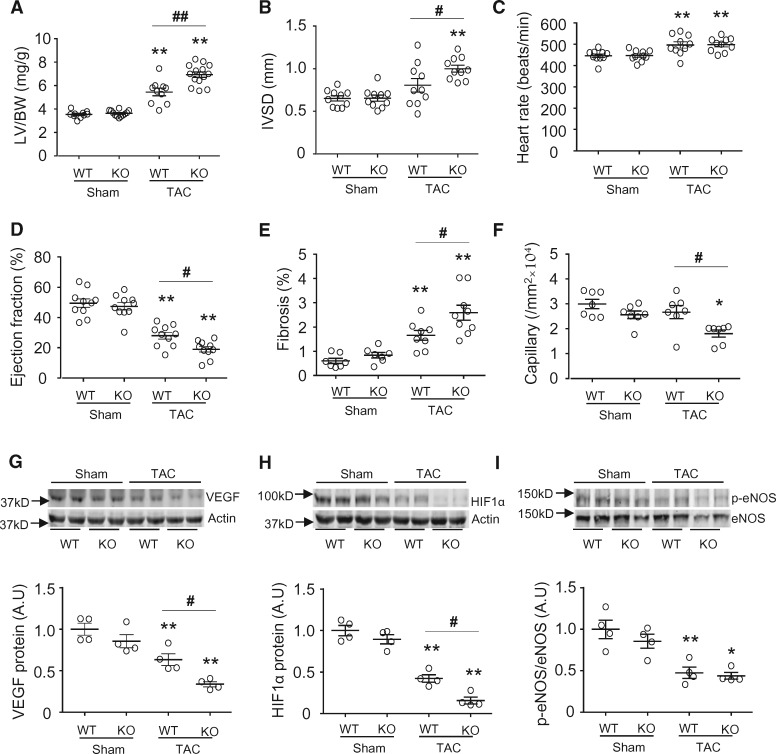
Nox4-null mice have exaggerated cardiac hypertrophy and dysfunction in response to TAC. (*A*) Left ventricle/body weight (LV/BW) ratio 2 weeks after TAC (*n* = 10–14/group). (*B*–*D*) Echocardiographic analysis of WT and Nox4KO mice subjected to 2 weeks TAC (*n* = 10/group). IVSD, interventricular septal diameter; EF, ejection fraction. (*E*–*F*) Mean data for myocardial interstitial fibrosis and capillary density (*n* = 7–9/group). (*G*–*I*) Protein levels of VEGF-A, HIF1α, p-eNOS, and total eNOS in LV of *Nox4*-null mice compared to WT. Representative immunoblots are shown at the top and mean data below (*n* = 4/group). VEGF, vascular endothelial growth factor; HIF1α, hypoxia-induced factor-1α; eNOS, endothelial nitric oxide synthase. *P < 0.05, **P < 0.01 vs. respective sham controls. ^#^*P* < 0.05, ^##^*P* < 0.01 vs. WT/TAC, two-way ANOVA with Tukey’s Multiple Comparison Test. All data are presented as mean ± SEM.

To establish whether the role of Nox4 is similar in response to TAC as previously observed during AAB, TAC was performed in global Nox4KO and matched WT mice. The trans-stenotic pressure gradient assessed by echo-Doppler was similar in Nox4KO and WT mice 1 day after TAC (42 ± 7 cf. 44 ± 8 mmHg), and there was no difference in perioperative mortality rate. After 2 weeks of TAC, Nox4KO mice developed significantly greater LV hypertrophy (LVH) and contractile dysfunction than WT mice, as assessed by the LV/body weight ratio, interventricular septal thickness in diastole and LV ejection fraction (EF) (*Figure**1**A, B*, and *D*). The extent of interstitial fibrosis was significantly higher and myocardial capillary density significantly lower in Nox4KO than WT mice after TAC (*Figure**1**E* and *F*). Previous work showed that the mechanisms underpinning Nox4-mediated preservation of capillary density involve HIF1α/VEGF signaling.^4^ Western blotting revealed that VEGF and HIF1α protein levels were decreased in heart tissue after TAC compared to sham, and that the decrease was significantly greater in Nox4KO mice than controls (*Figure**1**G* and *H*). TAC also resulted in a decrease in the levels of phosphorylated eNOS (p-eNOS), but this occurred to a similar extent in the Nox4KO and control groups. Taken together with our previous findings using chronic AAB,^4^ these results indicate that global Nox4 deletion in the mouse heart has a detrimental impact on myocardial capillarization, remodelling, and contractile function in response to chronic pressure overload, independent of the type of aortic constriction/banding model.

### 3.2 Cardiomyocyte vs. endothelial cell Nox4

The studies above used global Nox4KO mice, leaving open the possibility that the beneficial effects of Nox4 on myocardial capillary density and cardiac remodelling might be driven solely by endothelial cell Nox4. To specifically establish the roles of cardiomyocyte and endothelial cell Nox4, we generated Cardio-Nox4KO and Endo-Nox4KO mice.

**Figure 2:**
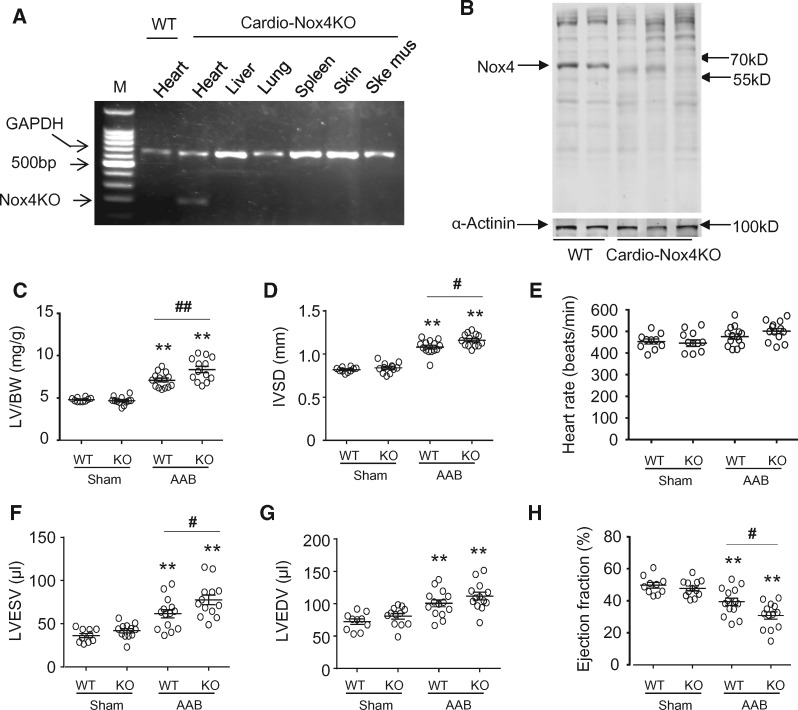
Cardio-Nox4KO mice have exaggerated cardiac dysfunction in response to pressure overload. (*A*) Representative image showing PCR products obtained using primers targeted against the Nox4 gene. Deletion of the first two exons in Nox4 KO tissues after 5 days of tamoxifen administration results in a truncated PCR product, which is observed only in the heart (third lane) – confirming a cardiac-specific knockout. (*B*) Nox4 protein levels in cardiomyocytes isolated from hearts of cardio-Nox4KO mice and wild-type controls. α-actinin was used as a loading control. (*C*) Left ventricle/body weight (LV/BW) ratio after 6 weeks AAB (*n* = 10–14/group). (*D*–*H*) Echocardiographic analysis of WT and Cardio-Nox4KO mice subjected to 6 weeks AAB (*n* = 10–14/group). IVSD, interventricular septal diameter; LVEDV, LVESV, LV end-diastolic and end-systolic volumes; EF, ejection fraction. **P < 0.01 vs. respective sham controls. ^#^*P* < 0.05, ^##^*P* < 0.01 vs. WT/AAB, two-way ANOVA with Tukey’s Multiple Comparison Test. All data are presented as mean ± SEM.

**Figure 3:**
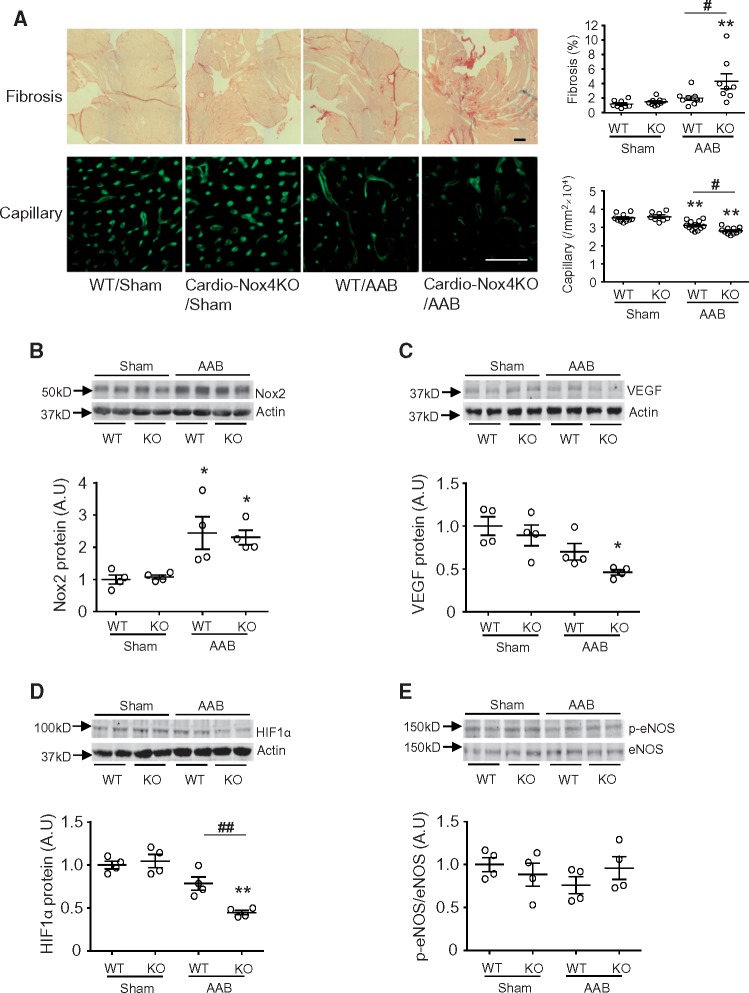
Cardio-Nox4KO mice display higher interstitial fibrosis and lower capillary density after 6 weeks abdominal aortic banding (AAB). (*A*) Representative LV sections from Cardio-Nox4KO mice and WT littermates stained for myocardial fibrosis and capillaries; scale bars 50 μm. Mean data are shown on the right (*n* = 8–12/group). (*B*) Nox2 protein levels in Cardio-Nox4KO and WT mice (*n* = 4/group). (*C*–*E*) Protein levels of VEGF-A, HIF1α, p-eNOS, and total eNOS in LV of Cardio-Nox4KO mice compared to respective WT. Representative immunoblots are shown at the top and mean data below (*n* = 4/group). *P < 0.05, **P < 0.01 vs. respective sham controls. ^#^*P* < 0.05, ^##^*P* < 0.01 vs. WT/AAB, two-way ANOVA with Tukey’s Multiple Comparison Test. All data are presented as mean ± SEM.

The cardiomyocyte specificity of Nox4 deletion in Cardio-Nox4KO mice was demonstrated by PCR (*Figure 2A*). Western blot analyses confirmed that cardiomyocytes isolated from Cardio-Nox4KO mice had a deletion of Nox4 protein (*Figure 2B*). Nox4 deletion in cardiomyocytes had no significant effect on basal cardiac structure or function (*Figures 2C–H*). Tamoxifen treatment itself had no effect on cardiac function (data not shown). We then studied the response to chronic pressure overload. Cardio-Nox4KO mice developed exaggerated cardiac hypertrophy and contractile dysfunction (*Figure 2C–H*) as well as increased interstitial fibrosis and a lower myocardial capillary density (*Figure 3A*) after chronic pressure overload as compared to their wild-type controls. Myocardial Nox2 protein levels were unaltered by Nox4 deletion in Cardio-Nox4KO mice and both the Cardio-Nox4KO and wild-type control showed a similar increase in Nox2 after AAB (*Figure 3B*). Investigation of potential mechanisms underlying the effects of Nox4 on capillary density revealed that the myocardial protein levels of VEGF and HIF1α were significantly lower in Cardio-Nox4KO mice than wild-type controls after pressure overload (*Figure**3**C* and *D*). However, there were no significant differences in p-eNOS among groups (*Figure 3E*).

**Figure 4:**
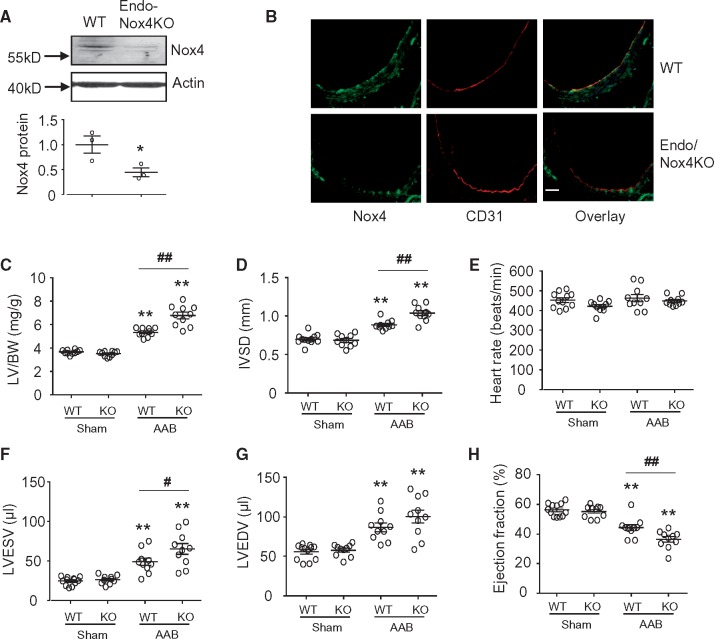
Endo-Nox4KO exhibit worse load-induced dysfunction than wild-type controls. (*A*) Nox4 protein expression in aorta of wild-type and endo-Nox4KO mice. *P < 0.05, n = 3, unpaired Student’s *t*-test. (*B*) Immunostaining for Nox4. Aortic sections stained for Nox4 (green) and CD31 (red) as an endothelial cell marker. Scale bars 50 μm. The yellow colour in the merged images in the right panels denotes co-localization. (*C*) Mean data for cardiac hypertrophy in terms of left ventricle weight/body weight ratio (LV/BW) (*n* = 10/group). (*D*–*H*) Echocardiographic analysis of WT and Endo-Nox4KO mice subjected to 6 weeks AAB (*n* = 10–12/group). IVSD, interventricular septal diameter; LVEDV, LVESV, LV end-diastolic and end-systolic volumes; EF, ejection fraction. **P < 0.01 vs. respective sham controls. ^#^*P* < 0.05, ^##^*P* < 0.01 vs. WT/AAB, two-way ANOVA with Tukey’s Multiple Comparison Test. All data are presented as mean ± SEM.

**Figure 5:**
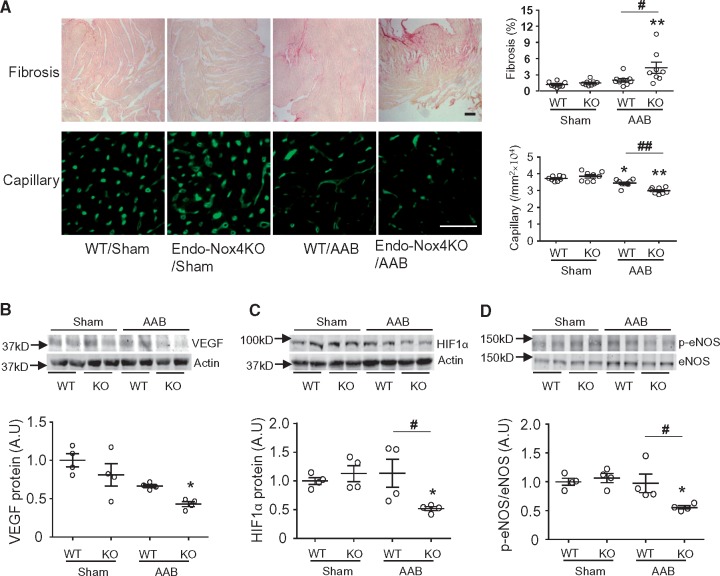
Myocardial interstitial fibrosis and capillary density in Endo-Nox4KO mice. (*A*) Representative LV sections from Endo-Nox4KO mice and WT littermates stained for myocardial fibrosis and capillaries; scale bars 50 μm. Mean data are shown on the right (*n* = 8–9/group). (*B*–*D*) Protein levels of VEGF-A, HIF1α, p-eNOS, and total eNOS in LV of Endo-Nox4KO mice compared to respective WT. Representative immunoblots are shown at the top and mean data below (*n* = 4/group). *P < 0.05, **P < 0.01 vs. respective sham controls. ^#^*P* < 0.05, ^##^*P* < 0.01 vs. WT/AAB, two-way ANOVA with Tukey’s Multiple Comparison Test. All data are presented as mean ± SEM.

The endothelial deletion of Nox4 in Endo-Nox4KO mice was confirmed by Western blot and immunohistochemical studies (Figure *4**A* and *B*), and had no effect on basal cardiac structure or function (*Figure 4C–H*). In response to chronic pressure overload, Endo-Nox4KO mice also developed exaggerated cardiac hypertrophy and contractile dysfunction (*Figure 4C–H*), increased interstitial fibrosis, and a lower myocardial capillary density (*Figure 5A*) than their wild-type controls. The myocardial protein levels of VEGF and HIF1α were reduced to a significantly greater extent after pressure overload in Endo-Nox4KO mice than controls (*Figure**5**B* and *C*), similar to the findings in Cardio-Nox4KO animals. In contrast to the Cardio-Nox4KO mice, however, Endo-Nox4KO animals showed a significant reduction in myocardial p-eNOS levels after AAB as compared to banded control mice (*Figure 5D*).

Overall, these results indicate that endogenous Nox4 in cardiomyocytes as well as endothelial cells is involved in preserving myocardial capillary density and limiting the detrimental effects of chronic pressure overload-induced cardiac remodelling.

## 4. Discussion

Whether Nox4 in the heart promotes adaptive responses or has detrimental effects in the context of chronic pressure overload has been debated. One suggestion to account for the divergent published data^4^^,^^6^ was that the responses might differ during TAC as compared to AAB. It is well recognized that TAC typically induces faster and more severe cardiac hypertrophy and dysfunction than AAB. In addition, AAB may be accompanied by renal hypoperfusion and activation of the renin–angiotensin system due to the more distal site of constriction. In the current study, 2 weeks of TAC induced an approximately 55% increase in LV mass and an >40% decrease in EF in WT mice. This compares with a similar increase in LV mass but a much smaller (23%) reduction in EF in the 6-week AAB model utilized in our previous study,^4^ consistent with AAB being a less severe model. Nevertheless, in the current study, we found that global Nox4KO mice still developed greater LVH, fibrosis, and systolic dysfunction and had a lower capillary density than WT after TAC, consistent with our previous results with AAB. It is important to note that a later study from the Sadoshima laboratory found that global Nox4 KO mice had exaggerated cardiac hypertrophy, dysfunction, and fibrosis as compared to WT controls after TAC^22^ (shown in online figure XVI in that paper). Therefore, both our and the Sadoshima laboratory find that the response to TAC is worse in global Nox4KO mice than WT controls, indicating that Nox4 is beneficial in this setting. It should also be noted that the TAC procedure used by the Sadoshima lab^6^ was significantly more severe than our procedure,^4^ the trans-stenotic pressure gradients are ∼70 vs. ∼40 mmHg, the extent of LVH is ∼60% vs. 55%, and the reduction in EF is ∼60% vs. 40%. Despite these differences in severity of TAC, global Nox4 deletion was detrimental in both settings. Taken together, the current results along with these previously published studies clearly show that a global deletion of Nox4 aggravates the response of the mouse heart to chronic pressure overload, regardless of the site and severity of aortic constriction.

Having ruled out the site of aortic constriction as the reason for the discrepant results of the studies investigating the role of Nox4 during chronic pressure overload, we next investigated the hypothesis that cardiomyocyte Nox4 is detrimental but endothelial Nox4 is beneficial. We directly tested this by generating Cardio-Nox4KO and Endo-Nox4KO mouse models to study their response to pressure overload. Furthermore, in order to avoid any possible confounding developmental effects of cardiomyocyte Nox4 deletion, we employed a tamoxifen-inducible Cardio-Nox4KO model so that Nox4 levels could be reduced in adult mice. A key finding from this experiment is that the cardiomyocyte-specific deletion of Nox4 is detrimental during chronic pressure overload, similar to our previous findings in global Nox4 knockout mice. Furthermore, these detrimental effects are associated with a significantly lower myocardial capillary density in Cardio-Nox4KO mice during pressure overload, consistent with our previous data in a cardiomyocyte-specific Nox4 gain-of-function model showing that myocardial Nox4 preserves capillary density.^4^ Therefore, endogenous cardiomyocyte Nox4 is indeed capable of mediating protective effects in the chronically overloaded heart.

Our studies in Endo-Nox4KO mice demonstrate that endothelial Nox4 as well as cardiomyocyte Nox4 contributes to cardiac adaptation to chronic pressure overload. While the beneficial effect of Nox4 in the vasculature has recently been reported,^7^^,^^10–12^ the current study is to our knowledge the first to demonstrate a protective role of endothelial Nox4 in the chronically stressed heart. The finding that capillary density was compromised in Endo-Nox4KO mice during pressure overload suggested that a likely mechanism underlying the beneficial effects of endothelial Nox4 may be via enhanced angiogenesis (similar to the mechanism identified in our prior study in global Nox4 KO mice^4^). Previous studies (including our own study in the heart) suggested that an enhancement of HIF signalling and/or endothelial nitric oxide synthase signalling may be involved in the pro-angiogenic effects of Nox4.^7–9^ We therefore assessed changes in HIF1α, VEGF, and p-eNOS (as a marker of eNOS activation) in all three models that were studied. We consistently found significant decreases in HIF1α and VEGF protein levels in overloaded Nox4-deficient animals as compared to controls in all models, i.e. Cardio-Nox4KO, Endo-Nox4KO, and global Nox4KO mice, strongly suggesting that a common general mechanism underlies the Nox4-dependent preservation of myocardial capillary density. Interestingly, a Nox4-dependent change in p-eNOS was observed only in the Endo-Nox4KO model, suggesting that this may be an additional endothelial-specific mechanism. Given the similar effects on myocardial capillary density and similar extent of change in contractile function observed in Cardio-Nox4KO and Endo-Nox4KO mice subjected to AAB, it is possible that functional communication between endothelial cells and cardiomyocytes^23^—involving Nox4 in both cell types—is important in cardiac adaptation to chronic pressure overload. Consistent with this idea, it was previously shown that Nox4 enhances the paracrine release of pro-angiogenic VEGF from cardiomyocytes^4^ and is also involved at endothelial cell level in promoting angiogenesis.^7^^,^^8^

The current work clearly demonstrates that both cardiomyocyte and endothelial cell Nox4 mediate protective effects during chronic hemodynamic overload. So what explains the findings initially reported by Kuroda et al^6^ that cardiomyocyte-specific Nox4KO was beneficial? In the setting of cardiomyocyte-specific overexpression of Nox4, it could be envisaged that an excessively high level of Nox4 expression and/or possible mislocalization of the protein could lead to detrimental effects.^24^ The divergent results in a knockout model are more difficult to explain. Our Nox4KO mouse was generated by targeting the translation initiation site and the first two exons of the gene, which leads to a complete absence of Nox4 protein.^4^ The knockout mouse studied by Kuroda et al^6^ was generated by targeting exon 9, which could potentially result in a truncated protein that may have some biological effects. The numbers of mice that were studied for response to TAC in their study were very small—only three to four per group. Kuroda et al^6^ reported that Nox4 was located in the mitochondria, but we found it to be located predominantly in the endoplasmic reticulum^4^ and other work failed to find evidence of Nox4 in cardiac mitochondria after careful preparation of pure mitochondria.^25^ In fact, later work from the Sadoshima laboratory reported that Nox4 was located in the nucleus and the endoplasmic reticulum.^15^^,^^22^ As discussed earlier, the TAC model used by Kuroda et al^6^ is significantly more severe than our model, and led to a >30% initial mortality. Even though the studies in global Nox4KO mice suggest that TAC severity may not be important (because both labs find global Nox4 deletion to be detrimental), it is conceivable that severe TAC could have different effects on cardiomyocyte Nox4 than more moderate TAC. We did not study very severe TAC (resulting in >30% early mortality) in the current study because it may have limited pathophysiological relevance and was precluded by animal welfare considerations.

Based on the current results, at least some of the controversies regarding Nox4 in the haemodynamically overloaded heart can be resolved. It can unequivocally be concluded that Nox4 both in cardiomyocytes and endothelial cells is able to mediate protective effects in the haemodynamically overloaded heart. It is also clearly evident that Nox4 in global terms (as assessed by global Nox4 deletion) is beneficial in the heart under hemodynamic overload. It is theoretically possible that cardiomyocyte Nox4 might not be beneficial in a setting of very severe acute pressure overload (although still hard to envisage why it would be actively detrimental), but this is a setting with limited pathophysiological relevance. Overall, the current work indicates that the suggestion that Nox4 might be a suitable therapeutic target in the heart should be treated with great caution, especially considering that most therapeutic approaches (e.g. Nox inhibitors) involve small molecules that in principle affect Nox4 in all cell types. Finally, approaches to target Nox4 in other disease settings (e.g. cancer) should take into account the potential cardiac risks that might arise with such an approach, especially in subjects with pressure overload (e.g. hypertension).

## Funding

This work was supported by the British Heart Foundation (BHF RG/13/11/30384) and a Foundation Leducq Transatlantic Network of Excellence Award.

**Conflict of interest:** none declared.
